# Live Detection of Neural Progenitors and Glioblastoma Cells by an Oligothiophene Derivative

**DOI:** 10.1021/acsabm.3c00447

**Published:** 2023-08-30

**Authors:** Shirin Ilkhanizadeh, Aileen Gracias, Andreas K.O. Åslund, Marcus Bäck, Rozalyn Simon, Edel Kavanagh, Bianca Migliori, Christina Neofytou, Sven Nelander, Bengt Westermark, Lene Uhrbom, Karin Forsberg-Nilsson, Peter Konradsson, Ana I. Teixeira, Per Uhlén, Bertrand Joseph, Ola Hermanson, K. Peter R. Nilsson

**Affiliations:** † Department of Neuroscience, Karolinska Institutet, Stockholm 171 77, Sweden; ‡ IFM, Department of Chemistry, Linköping University, Linköping 581 83, Sweden; § Institute of Environmental Medicine, Karolinska Institutet, Stockholm 171 77, Sweden; ∥ Department of Immunology, Genetics and Pathology, and Science for Life Laboratory, Rudbeck Laboratory, Uppsala University, Uppsala 751 85, Sweden; ⊥ Department of Medical Biochemistry and Biophysics, Karolinska Institutet, Stockholm 171 77, Sweden

**Keywords:** bioelectronics, progenitor, brain tumor, methylation, p75NTR

## Abstract

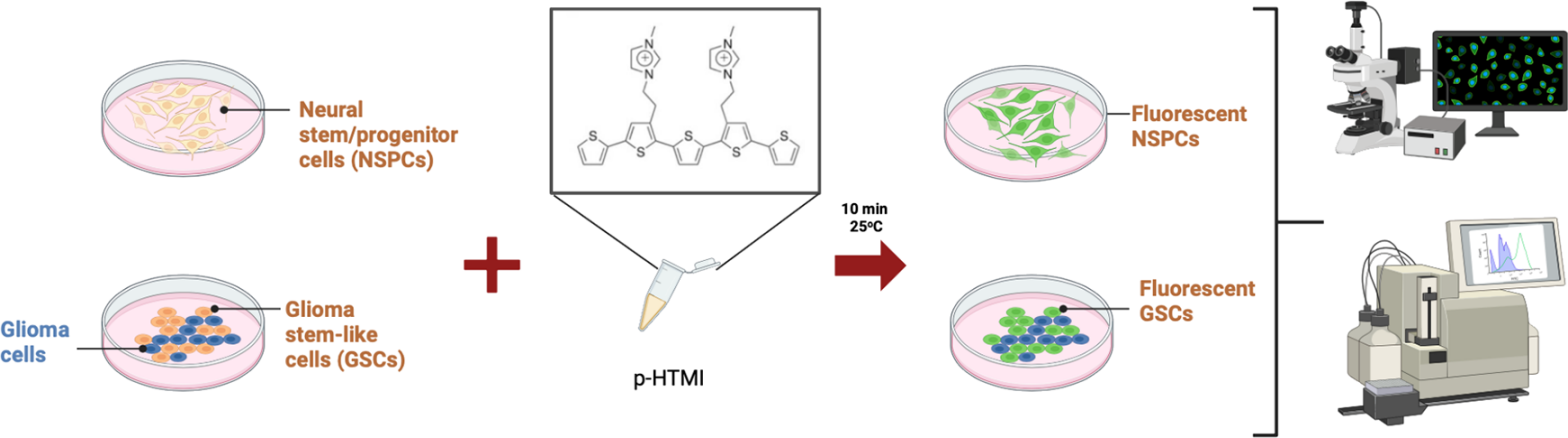

There is an urgent need for simple and non-invasive identification of live neural stem/progenitor cells (NSPCs) in the developing and adult brain as well as in disease, such as in brain tumors, due to the potential clinical importance in prognosis, diagnosis, and treatment of diseases of the nervous system. Here, we report a luminescent conjugated oligothiophene (LCO), named p-HTMI, for non-invasive and non-amplified real-time detection of live human patient-derived glioblastoma (GBM) stem cell-like cells and NSPCs. While p-HTMI stained only a small fraction of other cell types investigated, the mere addition of p-HTMI to the cell culture resulted in efficient detection of NSPCs or GBM cells from rodents and humans within minutes. p-HTMI is functionalized with a methylated imidazole moiety resembling the side chain of histidine/histamine, and non-methylated analogues were not functional. Cell sorting experiments of human GBM cells demonstrated that p-HTMI labeled the same cell population as CD271, a proposed marker for stem cell-like cells and rapidly migrating cells in glioblastoma. Our results suggest that the LCO p-HTMI is a versatile tool for immediate and selective detection of neural and glioma stem and progenitor cells.

## Introduction

Small probes specific to a distinct biomolecule or structural element have proven useful for recording biological events. In this regard, luminescent conjugated oligo- and polythiophenes (LCOs and LCPs) have been utilized as target-specific chameleons that change their emission depending on the structural motif of a distinct target molecule even in a complex environment, such as tissue.^1−4^ LCOs and LCPs contain a repetitive flexible thiophene backbone, and a conformational restriction of the thiophene rings upon interaction with a biomolecule leads to a distinct optical fingerprint of the dyes. LCOs and LCPs display intrinsic fluorescence properties and thus allow us to use a variety of imaging techniques and detection modes, i.e., full excitation/emission spectra, and fluorescence decay time, enhancing the toolbox of fluorescent ligands for optical assignment of disease-associated protein aggregates.^6^ Previously, chemically defined anionic pentameric LCPs have been reported as amyloid-specific ligands for optical detection of pathogenic protein aggregates *in vivo.*(5,6) LCPs have been shown to detect such aggregates in tissue sections from transgenic mouse models or patients, as well as in living cells and animals.^5,6^ Perhaps most importantly, when used for live imaging, LCPs and LCOs have been shown to not display any severe cellular or acute toxicity.^5^ Hence, LCOs and LCPs hold great promise also as potential tracers that can be utilized for clinical imaging in the future.^6^

Stem and progenitor cells derived from the developing and adult brain (NSPCs) and stem/progenitor cell-like populations derived from glioblastoma (GBM) tumors have caught special attention due to the need for new approaches in diagnosis, treatment, and possible cures for diseases of the nervous system.^7−9^ GBM is an aggressive type of nervous system tumor with a mean survival time of 12–14 months, and the survival time has remained low, despite major efforts.^10^ At least one subpopulation of progenitors with stem cell-like properties can be derived from GBM tumors, and these cells are commonly referred to as glioblastoma-derived stem cell-like cells (GSCs).^11−16^ GSCs seem to escape conventional irradiation treatment, chemotherapy, and surgery^10−13^ and correlate with poor patient prognosis as well as the risk of relapse,^15,16^ and it is therefore urgent to develop novel approaches for reliable detection of these and other cell types in GBM. While genetic approaches have proven successful in identifying and deciphering progenitors and stem cells in the healthy nervous system,^17,18^ simple and reproducible techniques to rapidly identify and detect live NSPCs and GBM cells without invasive or genetic modulation are desirable.^19^ Most commonly, either antibodies or tools like green fluorescent protein (GFP) are used for detection of specific stem cell types, but these modes have several limitations for detection of live cells as they often are time-consuming, require secondary amplification, and manipulate the cells or organism.

Herein, we investigate the use of a library of LCOs with distinct side-chain functionalization as molecular probes for the specific identification and detection of NSPCs and GBM-derived cells in real time.

## Experimental Section

For a detailed description of the synthesis of p-HTMI and related LCOs, please see the Supporting Information.

### Cell Culture

Rat embryonic neural stem and progenitor cells (NSPCs) were derived and cultured as previously described.^17−19^ Briefly, NSPCs were harvested from the cerebral cortices of E15.5 embryos of timed pregnant Sprague Dawley rats. Cells were mechanically dissociated and plated on previously coated tissue culture plates. NSPCs and C6 (ATCC CCL-107)-derived stem cell-like cells were expanded in N2 medium supplemented with 10 ng/mL FGF2 (R&D Systems) until 80% confluency was reached. Animals were treated in accordance with institutional and national guidelines (ethical permit nos. N310/05, N79/08, and N284/11).

### Staining of Cells with LCOs

In general, the LCOs were dissolved in deionized water to a final concentration of 1 mg/mL and administered at a dilution of 1:500 (1:10–1:10,000 were tested), directly to each well, and the detection was done after 10 min in a fluorescent microscope or FACS. p-HTMI generated fluorescence at a wavelength common to green fluorescent proteins.

### Two-Photon Microscopy of NSPCs Stained with p-HTMI

The emission wavelength of p-HTMI was characterized with a two-photon microscope. NSPCs were grown in 35 mm plates (40,000 cells/plate) and treated with 10 ng/mL FGF2 for 48 h. Prior to staining the cells, the medium was changed to DMEM:F12 medium without phenol red (Invitrogen) in order to eliminate background signals. CellTracker (Invitrogen) was administered together with p-HTMI (stock solution of 1 mg/mL in deionized water, diluted to 1:500), in order to be able to find living cells, resulting in a double staining in red (CellTracker) and green (p-HTMI).

### Immunocytochemistry

The plates were first rinsed once in PBS and then fixed in 10% formaldehyde for 20 min. The formaldehyde was aspirated, and the plates were washed 3 times, 5 min each, in PBS/0.1% Triton-X 100. The plates were then incubated with respective primary antibodies in PBS/0.1% Triton-X 100/1% BSA overnight at 4 °C. The samples were then washed 6 times, 5 min each, in PBS/0.1% Triton-X 100. Secondary antibodies (1:500) in PBS/0.1% Triton-X 100/1% BSA were incubated with the samples at room temperature for 1 h. The samples were then washed 3 times in PBS and mounted with Vectashield containing DAPI. Fluorescent images were acquired using a Zeiss Axioskop2 coupled to an MRm (Zeiss) camera at 10×, 20×, and 40× magnifications with Axiovision software. The primary and secondary antibody sources and dilutions were as follows: mouse anti-nestin from BD Biosciences Pharmingen (1:500), mouse monoclonal antineuronal class III β-tubulin (TuJ1) from Nordic Biosite (1:500), mouse monoclonal anti-a-smooth muscle actin (SMA) from Sigma (1:1000), rabbit polyclonal anti-CD133 from Invitrogen (1:500), and rabbit polyclonal antiglial fibrillary acidic protein (GFAP) from DAKO (1:500). Species-specific Alexa-488 and Alexa-594-conjugated secondary antibodies were used as appropriate and were obtained from Molecular Probes (1:500).

### Staining of Rat NSPCs

NSPCs were plated in a 6-well plate (40,000 cells/well) in serum-free DMEM:F12 (Invitrogen) with supplements and 10 ng/mL FGF2 (R&D systems) for 24 h prior to further stimulation. Cells were then stimulated with 10 ng/mL FGF2, 10 ng/mL CNTF (R&D systems), 10% FBS (Invitrogen), 1 mM VPA (Sigma), or without added factors (N2 medium) for 3 days. Addition of soluble factors was carried out every 24 h, and media was changed every 48 h. The LCOs were dissolved in deionized water to a final concentration of 1 mg/mL and administered at a dilution of 1:500, directly to each well, and the detection was done after 10 min in a fluorescent microscope. p-HTMI generated fluorescence at a wavelength common to green fluorescent proteins. A strong green signal was obtained in undifferentiated immature stem cells, accumulated in the cytoplasm of the cells, whereas differentiated and more mature cells displayed a significantly lower or no signal. Neural stem cells were also treated with 10 ng/mL BMP4 and 10 ng/mL Wnt3a (R&D Systems) for 14 days and then stained with p-HTMI.

### Staining of Rat C6 Glioma

C6 (ATCC CCL-107) glioma is a rat cell line used as a model system for glioma cells. The cells were grown in DMEM medium (Invitrogen) supplemented with 10% FBS. For the purpose of maintenance, the C6 glioma cells were grown in 75 cm^2^ flasks. Prior to experiments, the cells were split and plated (40,000 cells/well) in a 6-well plate. HEK-293 (ATCC CRL-1573), COS-7 (ATCC CRL-1651), and CV-1 (ATCC CCL-70) fibroblast cell lines were cultured according to the supplier’s recommendations. The primary and secondary antibody sources and dilutions were as follows: mouse anti-nestin from BD Biosciences Pharmingen (1:500), mouse monoclonal anti-neuronal class III β-tubulin (TuJ1) from Nordic Biosite (1:500), and rabbit polyclonal anti-glial fibrillary acidic protein (GFAP) from DAKO (1:500). Species-specific Alexa-488 and Alexa-594-conjugated secondary antibodies were used as appropriate and were obtained from Molecular Probes (1:500).

### Staining of Rat C6 Glioma Cultured as NSPCs

C6 glioma cells were cultured with the same protocol as for NSPCs, on plates precoated with poly-l-ornithine and fibronectin, and then grown in N2 medium with supplements. The cells were plated in a 6-well plate (40,000 cells/well) in serum-free DMEM:F12 (Invitrogen) with supplements and 10 ng/mL FGF2 (R&D systems) for 24 h prior to further stimulation. Cells were then stimulated with 10 ng/mL FGF2, 10 ng/mL CNTF (R&D systems), 10% FBS (Invitrogen), 1 mM VPA (Sigma), or without added factors (N2 medium) for 3 days. Addition of soluble factors was carried out every 24 h, and media was changed every 48 h. p-HTMI was administered, from a stock solution of 1 mg/mL at a dilution of 1:500, directly to each well, and the detection was done after 10 min in a fluorescent microscope. p-HTMI generated chemoluminescence at a wavelength common to green fluorescent proteins.

### RT-qPCR

Total RNA was extracted from cells using RNeasy (Qiagen) and contaminating DNA digested using an RNase-free DNase kit (Qiagen). cDNA was synthesized using 200 ng of total RNA using a high-capacity cDNA reverse transcription kit (Applied Biosystems). A 1:25 dilution of the cDNA was used for real-time PCR. Platinum SYBR Green qPCR Supermix UDG (Invitrogen) was used for real-time PCR analysis with a 7500 PCR system (Applied Biosystems). Primers (MWG Biotech) are available on request.

### FACS Sorting of NSPCs Stained with p-HTMI

NSPCs were grown in 35 mm plates (40,000 cells/plate) and were treated with 10 ng/mL FGF2 for 48 h. Prior to staining the cells, the medium was changed to DMEM:F12 medium without phenol red (Invitrogen) in order to eliminate background signals. Plates were incubated with p-HTMI (stock solution of 1 mg/mL in deionized water, diluted 1:500) for 10 min and then incubated with Hanks' solution for 5 min. The cells were then scraped and run through a FACS machine. Analysis was carried out on an FACSCalibur flow cytometer equipped with CellQuest software (Becton Dickinson).

### Cell Culture and FACS Sorting of Glioblastoma-Derived Stem Cell-like Cells (GCs) and U-87MG with p-HTMI, CD133, CD44, CD271, PI, and Annexin V

Patient-derived GBM cells (U3034MG, U3088MG, and U3031MG) or glioma cells (GCs) were grown in 6-well plates (50,000 cells/well), coated with poly-l-ornithine (15 μg/mL) and laminin (10 μg/mL), and grown to a confluency of 80%. Cells were expanded in medium containing neurobasal, DMEM:F12 glutamax, media supplements, and EGF and FGF (10 μg/mL, 1:1000) every 72 h. The plates were incubated with p-HTMI (stock solution of 1 mg/mL in deionized water, diluted 1:500) for 10 min, then incubated with Accutase for 7 min, and resuspended in cell culture medium. Cells were spun down at 1500 rpm for 5 min. Further, they were incubated with binding buffer (BSA, 0.5 M EDTA, and PBS) (100 μL/sample) and CD133-APC (130-090-826 Mitenyi Biotec) (10 μL/sample), incubated for 10 min at 4 °C, and washed with PBS prior to FACS analysis. Cells were incubated with CD44 (45-0441 eBioscience) diluted in binding buffer and used at a concentration of 1:10,000. The same antibody conditions as CD133 were used for CD44 i.e. 100 μL of binding buffer, incubated for 10 min at 4 °C, and washed with PBS prior to FACS analysis. Cells were also double stained for p-HTMI and CD271 (560834 BD Pharmingen), 5 μL/sample with the same antibody conditions as CD133. U-87MG cells were expanded as previously described^26^ and incubated with the same conditions of p-HTMI and CD133 and CD44 as described for GCs. To verify cell necrosis, lysis buffer was added with 5 μL of PI per sample, and for cell apoptosis, cells were incubated with 100 μL of 1X annexin V binding buffer and 5 μL of APC annexin V per sample (561012 BD Pharmingen) and incubated for 15 min. The analysis was carried out on an FACS LSRII flow cytometer equipped with FACSDiva software. Patient tissue collection and use were in accordance with ethical permit EPN Uppsala 2007/353 and its addendum Oct. 28, 2013.

## Results

In order to characterize the library of LCOs in an NSPC context, we used several very well-established differentiation protocols to generate a variety of cell types from NSPCs derived from embryonic rat cortices to compare with the undifferentiated cells. This NSPC protocol, based on fibroblast growth factor 2 (FGF2) treatment, is well-characterized and widely used^20^ and has been shown to contain ≫99% cells with the ability to differentiate into neurons, astrocytes, and oligodendrocytes.^20−22^ Interleukin-6-related cytokines such as the extrinsic factor ciliary neurotrophic factor (CNTF) induces astrocytic differentiation, fetal bovine serum (FBS) induces mesenchymal differentiation (smooth muscle cell-like), and a combination of BMP4/Wnt3a induces neuronal and astrocytic differentiation.^23^

**Figure 1:**
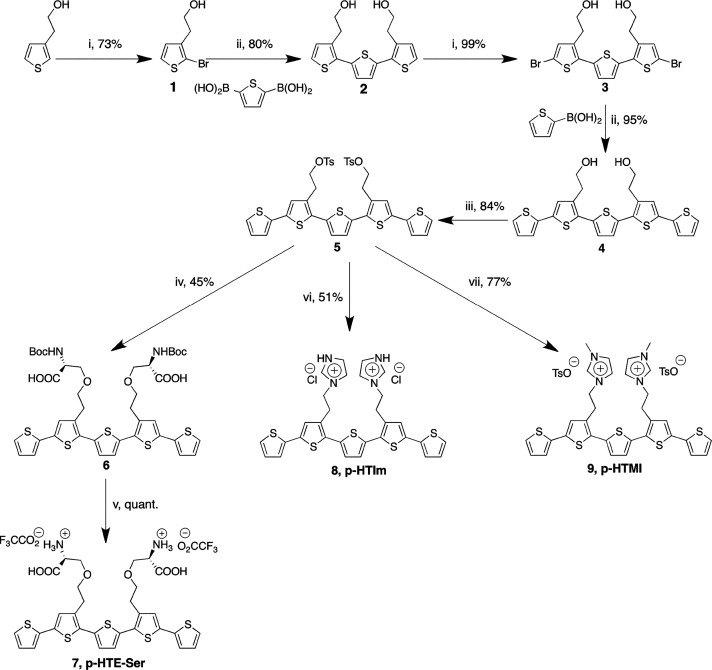
Synthesis of p-HTMI. Reagents and conditions: (i) *N*-bromosuccinimide, DMF, −15 °C; (ii) PEPPSI-IPr, K_2_CO_3_, toluene/methanol (1:1), 75 °C; (iii) *p*-toluenesulfonyl chloride, CHCl_3_, pyridine; (iv) methylimidazole, acetonitrile, 75 °C; (v) imidazole, acetonitrile; (vi) Boc-l-Ser-OH, K_2_CO_3_, DMF, 50 °C; (vii) TFA, CH_2_Cl_2_.

In a screen with various LCOs, we identified p-HTMI (Figure 1 and Figures S1 and S2) as a potential marker for NSPCs. Within 10 min after application of p-HTMI by simply pipetting into the cell culture medium, fluorescence at a wavelength common to green fluorescent proteins was generated in undifferentiated embryonic rat NSPCs (Figure S2b). Two-photon microscopy revealed that a strong fluorescent signal accumulated in the cytoplasm of the cells, thus indicating that the probe had efficiently crossed the cell membrane (Figure S2d and Movie S1). Simultaneous detection of a chloromethyl derivative detecting all live cells with red fluorescence (CellTracker) demonstrated that p-HTMI-stained cells were viable. The red and green fluorescence could be detected in parallel in the same cells, and thus, p-HTMI allowed double labeling of single cells (data not shown, see further below). The differentiated and more mature cells displayed a significantly lower or no signal (Figure S2b), and testing of a long series of various cells, including several commonly used cell lines (HEK-293, CV-1, COS-7, etc.), mesenchymal stem cells, or embryonic stem cells (see further below), did not result in any staining by p-HTMI. In contrast, the variant LCO p-HTE-Ser with a different side-chain functionality (Figure S1) displayed no signal in undifferentiated NSPCs, whereas a weak staining was observed in fully differentiated smooth muscle cells and mature astrocytes (Figure S3). Anionic LCPs and LCOs (PTAA, p-FTAA, p-HTAA, and h-HTAA (Figure S1)) previously reported to specifically detect amyloid structures^24,25^ displayed no cell-specific signal in undifferentiated NSPCs (data not shown).

As p-HTMI is functionalized with methylated imidazole moieties (Figure 1 and Figure S2a) resembling the side chain of histidine/histamine, we asked whether the specificity of the NSPC staining was dependent on this distinct side chain functionalization. We therefore generated pentameric LCOs with imidazole moieties lacking the methylation, p-HTA-His and p-HTIm (Figure S1). Interestingly, we found that these molecules did not detect the NSPCs tested above (Figure S2c and data not shown), indicating that the methylation of the imidazole moieties is a crucial factor for the efficiency and, importantly, specificity of the detection. Notably, a previously reported polydisperse LCP with methylated imidazole moieties attached to all thiophene units, PTMI^26^ (Figure S1), did not stain the NSPCs either, suggesting that the length of the thiophene backbone and the positioning of the methylated imidazole moieties are additional important parameters to obtain the specific staining of the NSPCs.

Fluorescence-activated cell sorting (FACS) is commonly used to quantitatively record fluorescent signals from individual cells and to separate cells based on fluorescence. We stained undifferentiated FGF2-treated NSPCs with either p-HTMI or p-HTE-Ser. Using FACS, we then sorted unstained cells, p-HTMI-stained cells, and p-HTE-Ser-stained cells. The cells stained with p-HTE-Ser displayed fluorescence comparable to the unstained cells, and this staining was thus considered background fluorescence (Figure S3). p-HTMI-stained cells, however, displayed a clear fluorescence peak, not overlapping with background fluorescence levels (Figure S4). In order to check for cell-to-cell leakage of the molecules, cells separately stained with p-HTMI and p-HTE-Ser were mixed and sorted with FACS. Two distinct fluorescence peaks were obtained, showing that cross contamination of the molecules did not occur (Figure S4). Taken together, these results verify that p-HTMI selectively detects undifferentiated embryonic NSPCs *in vitro*.

We next wanted to test whether the observed p-HTMI staining was a result of the treatment of the cells rather than the NSPC phenotype. We therefore used mouse embryonic stem cells (ESCs) or NSPCs derived from these cells by using an established retinoic acid-based protocol clearly distinct from the NSPC-expanding protocol used hitherto.^27,28^ Intriguingly, while p-HTMI only stained a small fraction of ESCs (Figure S5a), we found staining similar to that of embryonic brain-derived NSPCs in the ESC-derived NSPCs at an equivalent efficiency (Supporting Information, Figure S5b). This result indicated that p-HTMI is labeling embryonic or embryonic-like NSPCs and not cells cultured with a particular protocol. In addition, p-HTE-Ser labeled >90% of the ES cells but did not show any staining in the ESC-derived NSPCs (Figure S5a and data not shown).

**Figure 2:**
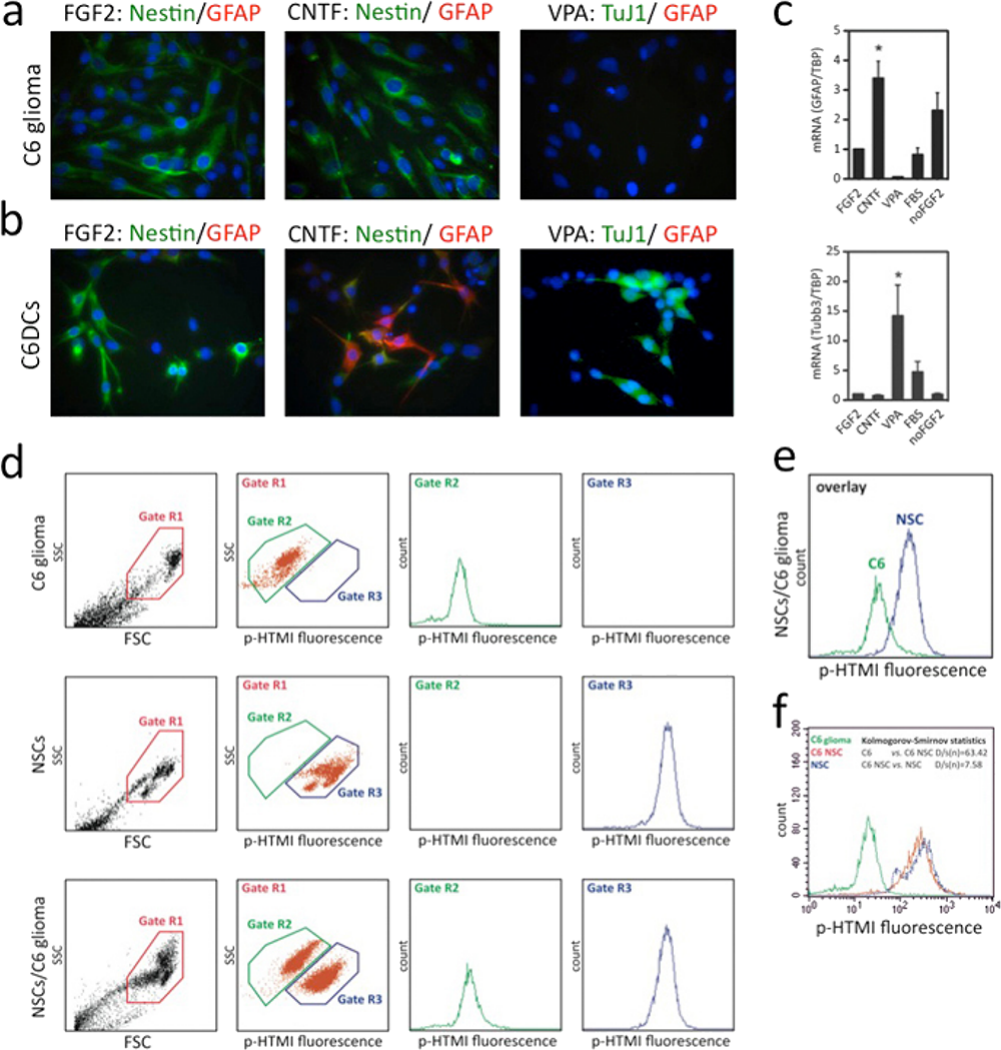
p-HTMI specifically detects glioma-derived stem-like cells by FACS. (a) Conventionally cultured C6 glioma cells are nestin-positive but stain negative for differentiation markers, also after treatment with CNTF or VPA. (b,c) C6 glioma cells cultured in 2D layers in the presence of FGF2 respond to CNTF and VPA, and increased numbers of GFAP-positive cells and TuJ1-positive cells (b), along with increased mRNA levels (c), are detected after administration of CNTF and VPA, respectively. (d–f) Cell sorting by FACS demonstrates that p-HTMI specifically and selectively detects glioma-derived stem-like cells and neural stem cells but not glioma cells.

Glioblastoma (GBM) is a common and aggressive type of brain tumor in adult humans.^8^ Rat C6 glioma can be considered an experimental model system for studying GBM. C6 glioma cells do not normally respond efficiently to differentiation-inducing factors, which was confirmed by our experiments (Figure 2a). We therefore applied the protocol for embryonic rat NSPCs to C6 glioma cells to see if the differentiation potential could be affected. Grown in the presence of FGF2 on fibronectin-coated plates and in N2 medium with supplements, C6 glioma cells grown as NSPCs (referred to as C6DCs) formed uniform cultures of nestin-positive cells (Figure 2b). In the C6DCs treated with CNTF, clear signs of increased astrocytic differentiation were detected (Figure 2b), and valproic acid (VPA) induced differentiation into neuronal-like, TuJ1-positive cells (Figure 2b) as previously shown for embryonic rat NSPCs.^29^ In addition, the gene expression of *Gfap* and *Tubb3* was elevated upon CNTF and VPA treatment (Figure 2c). Thus, rat C6 glioma cells grown in similar conditions as embryonic rat NSPCs become responsive to external factors and obtain at least a subset of characteristics of neural stem cells.

Various studies have suggested that 1–4% of the conventionally cultured C6 glioma cells share characteristics of cancer stem cells.^30^ We administered p-HTMI to regularly cultured C6 glioma cells and noted that this resulted in staining of around 1–2% of the cells (Figure 2d). In contrast, administration of p-HTE-Ser resulted in staining of >95% of the C6 glioma cells (Figure 2d). We next stained C6DCs grown in the presence of FGF2 using p-HTMI or p-HTE-Ser. Surprisingly, the C6DCs stained with p-HTMI now displayed a strong fluorescent signal, while p-HTE-Ser did not stain any cells (Figure 2d). FACS analysis confirmed these results and verified that a population of approximately 1% of regular C6 glioma cells is detected by p-HTMI (Figure 2d,e). This experiment further validated the ability of p-HTMI to distinguish between cell types as the vast majority of C6DCs and embryonic NSPCs could be clearly distinguished from conventionally cultured C6 glioma cells (Figure 2f).

**Figure 3:**
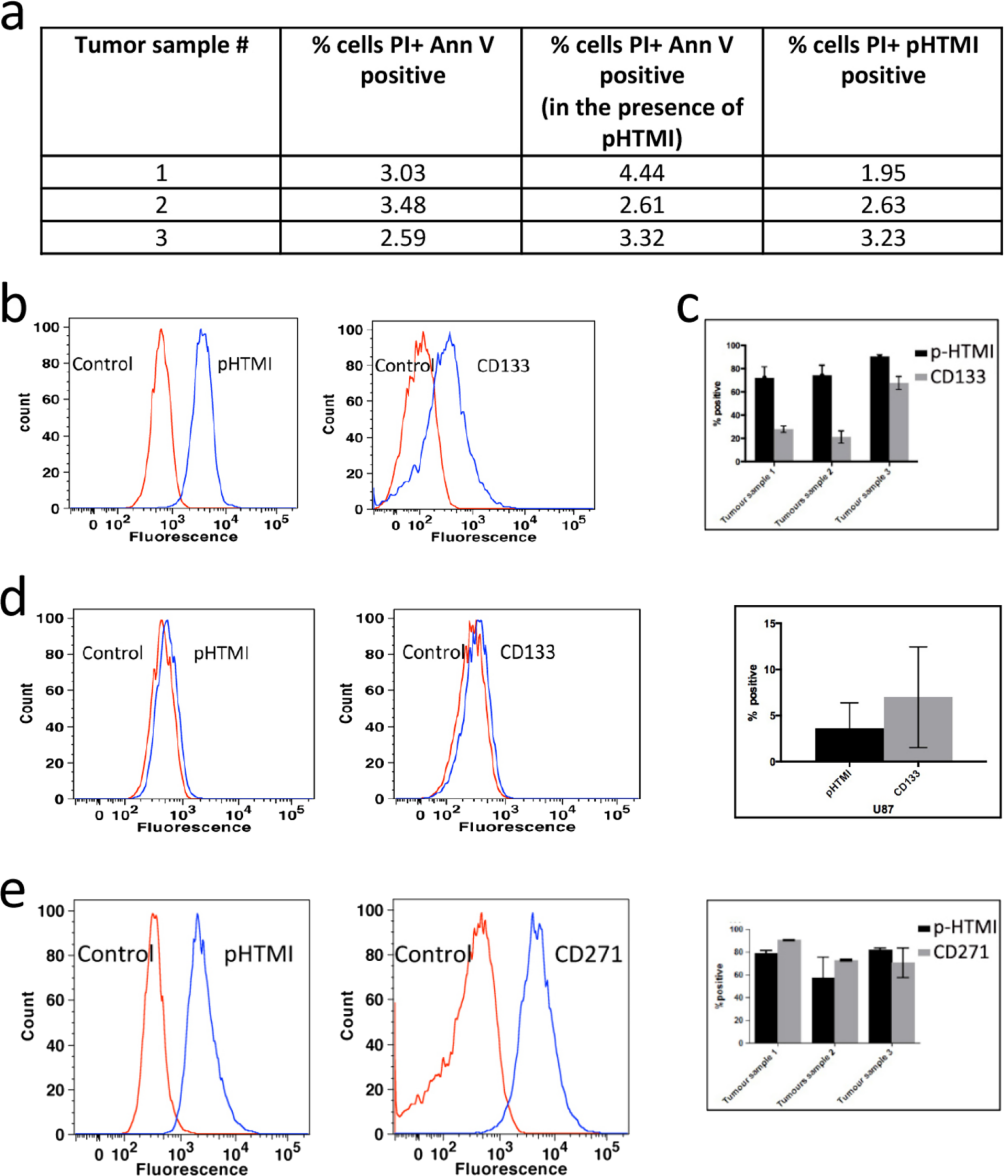
p-HTMI detects largely the same population of GBM cells as CD271/p75NTR. (a) Minimal cell necrosis and apoptosis in all three tumor samples in the presence of p-HTMI verified using propidium iodide (PI) and annexin V. (b,c) Comparison of p-HTMI and CD133-labeled GBM cells as assessed by fluorescence-activated cell sorting (FACS). (d) A small number of cells were detected by p-HTMI and CD133 in U-87MG glioma cells. (e) In a double-labeled cell population, p-HTMI and CD271 label largely the same population of cells in U3034MG, U3088MG, ad U3031MG nestin+/SOX2+ patient-derived GBM cells.

The obtained results from the C6 glioma cell line *in vitro* prompted an investigation, whereas the human GBM cells detected by p-HTMI shared characteristics of the so-called glioma stem-like cells, GSCs. We therefore used three previously characterized cell lines (U3034MG, U3088MG, and U3031MG) derived from human GBM expanded *in vitro*(31) from different individual patients with glioblastoma. These patient-derived cells have been shown to contain a high proportion of tumor-initiating cells (TICs), to stain positive for SOX2 and nestin, and show the ability to self-renew as well as differentiate into various neural cell fates.^31^ FACS experiments demonstrated that p-HTMI reproducibly detected between 70 and 90% of the cells in these three cultures within 10 min from application of the molecule (Figure 3b, c, and e). The transport of p-HTMI over the membrane seemed to be active, as no p-HTMI labeling could be detected in the GSCs at low temperatures (4–8 °C, data not shown). To assess whether p-HTMI exerted any effect on the cell survival of these cells, we investigated the cell death in the cultures of the treated cells but found no significant difference between control cultures and p-HTMI-treated cells when PI staining and the number of annexin V-positive cells were analyzed by FACS (Figure 3a).

We next aimed at elucidating the population of p-HTMI-positive cells detected in the GSC cultures by comparison with antibodies previously discussed to detect stem-like cells from various sources of cancer. CD133 or prominin is an antigen expressed by various neural progenitor populations, and it has been shown that depletion of CD133-positive cells from glioma cell populations decreases, but does not abolish, the tumor-initiating ability of transplanted glioma cells [e.g., ref (32)] In the GSC cultures examined, CD133 stained 20–70% of the cells in comparison to p-HTMI that stained 70–90% (Figure 3b,c). To investigate whether the higher number of cells detected by p-HTMI was merely the result of a less specific labeling, we compared labeling of the CD133 antibody and p-HTMI in the tumor cell line U-87MG, previously shown to contain a very small percentage of GSCs in cell culture.^33^ The number of cells detected by the CD133 antibody as well as p-HTMI was much lower in the U-87MG cultures, and in these cells, the number of cells detected by the CD133 antibody was higher than that of p-HTMI (Figure 3d). CD44 antibodies are under thorough investigation in various cancers for their ability to detect progenitor cells *in vivo* but may not be suitable for use *in vitro*. Here, the CD44 antibody detected basically all cells (>98%) in the three GC cultures as well as the U-87MG cell line and thus showed significantly less specificity than p-HTMI (Figure S6 and data not shown).

CD271, i.e., nerve growth factor receptor p75NTR, has been reported to be expressed by various types of progenitor cells in many organs and different tumor types. In accordance, it has been shown that the CD271 antibody selectively detects neural progenitors *in vitro* and *in vivo*.^34^ Interestingly, it has been shown that the CD271 antibody detects two subpopulations of cells in GBM, stem cell-like cells and the rapidly migrating cells.^35,36^ We therefore pursued a double staining experiment with p-HTMI and CD271 antibodies in GSC cultures and found by FACS that the cell populations in GSC cultures stained by p-HTMI and CD271 are vastly overlapping (Figure 3e). We conclude that p-HTMI-positive cells in GSCs are similar to those labeled by CD271, and thus, p-HTMI-stained cells may be subpopulations of stem cell-like cells and rapidly migrating GBM cells.

## Discussion

Here, we have demonstrated that p-HTMI is a novel molecular probe that specifically stains live progenitors derived from embryonic rodent brains and rodent and human glioma within 10 min by just mere application of the molecule to the cell culture and is thus representative of a more recent generation of smart molecules to be used for non-invasive, non-genetic live cell detection. It is noted that isolation of p-HTMI-positive cells from primary material followed by single cell analysis would be the next step to thoroughly investigate whether the p-HTMI-positive population is homogeneous or not, but we can conclude from the present results that the molecule detects neural stem and progenitor cells in development and glioblastoma. In addition, it will be of interest to pursue *in vivo* studies to investigate the possibility to use p-HTMI as a complementary tool to detect GSCs *ex vivo* in biopsies and in fluorescence-guided surgical resection of GBM. The CD271 antibody stains for cells derived from and present in various organs and is significantly less cell-specific than p-HTMI, and as any antibody, it only provides an indirect detection of the cells. Nevertheless, our results from co-staining with CD271 yet strengthen the verification of the specificity and versatility of p-HTMI. It is interesting to note that the intracellular localization of p-HTMI in stained cells seems to be concentrated to the cytoplasm, and in pilot experiments, it has been noted that p-HTMI labeling overlaps to a large extent with that of GM130, a protein located on the surface of the Golgi apparatus (B.M. and O.H., unpublished observations), and this may guide future studies on the intracellular target of the molecule.

## Conclusions

While neural stem and progenitor cells (NSPCs) hold great promise as therapeutic targets in various neurological diseases, stem cell-like cells in aggressive brain tumors, such as GBM, are potential targets for ablation. These cells, which we refer to as glioblastoma-derived stem cell-like cells, GSCs, seem to be key to the common relapse and poor prognosis associated with GBM. Yet, reliable and noninvasive markers to identify these cells are lacking. Here, we have used an alternative technology, LCOs, and unveiled that an oligothiophene, p-HTMI, detects NSPCs and GSCs *in vitro* from both rodents and humans, whereas other cell types are not stained by the molecule. Our findings unveil a potential new tool for surgeons and pathologists to use in the immediate future.

## Supporting Information Available

Synthesis; (Figure S1) structures of the LCOs evaluated; (Figure S2) oligothiophene p-HTMI specifically detecting embryonic neural stem and progenitor cells (NSPCs) *in vitro*; (Figure S3) different LCO, p-HTE-Ser, that does not stain NSPCs; (Figure S4) p-HTMI, but not p-HTE-Ser (P-HTES), efficiently and selectively labeling NSPCs in mixed cell populations; (Figure S5) p-HTMI detection of C6 glioma-derived and embryonic stem cell-derived NSPCs; (Figure S6) FACS analysis suggesting low specificity of CD44 in GSCs (PDF)
(Movie S1) Stacks of micrographs from two-photon microscopy that were assembled creating a 3D picture, demonstrating that the p-HTMI labeling was predominantly localized to the cytoplasm in NSPCs *in vitro* (MOV)

## Supplementary Material

mt3c00447_si_001.pdf

mt3c00447_si_002.mov
