# Supplementary material for: Live Detection of Neural Progenitors and Glioblastoma Cells by an Oligothiophene Derivative
Source: ACS Appl Bio Mater. 2023 Aug 30;6(9):3790–7. doi: 10.1021/acsabm.3c00447 (PMC10521023; doi:10.1021/acsabm.3c00447)
Supplement: Supplementary file 1 — mt3c00447_si_001.pdf [file mt3c00447_si_001.pdf]

SUPPORTING INFORMATION

Live detection of neural progenitor and glioblastoma cells by an oligothiophene derivative

Shirin Ilkhanizadeh[1,EC], Aileen Gracias[1,EC], Andreas K.O. Åslund[2,EC], Marcus Bäck[2,EC], Rozalyn Simon[2], Edel Kavanagh[3], Bianca Migliori[1], Christina Neofytou[1], Sven Nelander[4], Bengt Westermark[4], Lene Uhrbom[4], Karin Forsberg-Nilsson[4], Peter Konradsson[2], Ana I. Teixeira[5], Per Uhlén[5], Bertrand Joseph[3], Ola Hermanson§*[1], and K. Peter R. Nilsson§[2]

EC: These authors contributed equally to this study
§: These authors jointly directed this study

1: Department of Neuroscience, Karolinska Institutet, 171 77 Stockholm, Sweden.
2: IFM, Department of Chemistry, Linköping University, 581 83 Linköping, Sweden.
3: Institute of Environmental Medicine, Karolinska Institutet, 171 77 Stockholm, Sweden.
4: Department of Immunology, Genetics and Pathology, and Science for Life Laboratory, Rudbeck Laboratory, Uppsala University, 751 85 Uppsala, Sweden.
5: Department of Medical Biochemistry and Biophysics, Karolinska Institutet, 171 77 Stockholm, Sweden.

*: To whom correspondence should be addressed.
Professor Ola Hermanson
Department of Neuroscience
Biomedicum D7
Karolinska Institutet
SE17177 Stockholm
Sweden

Email: Ola.Hermanson@ki.se
Phone: +46-8-5248-7791, +46-76-118-7452.

Synthesis

Organic extracts were dried over anhydrous magnesium sulfate, filtered and concentrated *in vacuo* at 40 °C. 3-thiopheneethanol, 2-thiopheneboronic acid, thiophene-2,5-bisboronic acid, and PEPPSI-IPr([1,3-Bis(2,6-Diisopropylphenyl)imidazol-2-ylidene](3-chloropyridyl)palladium(II) dichloride) are commercially available from Sigma-Aldrich Co. NMR-spectra were recorded on a Varian instrument (^{1}H 300 MHz, ^{13}C 75.4 MHz). Chemical shift were assigned with the solvent residual peak as a reference according to Gottlieb *et al*. [20]. TLC was carried out on Merck precoated 60 F254 plates using UV-light (λ = 254 nm and 366 nm) and charring with ethanol/sulfuric acid/*p*-anisaldehyde/acetic acid 90:3:2:1 for visualization. Flash column chromatography (FC) was performed using silica gel 60 (0.040-0.063 mm, Merck). Gradient HPLC-MS was performed on a Gilson system (Column: Waters X-Bridge C-18 or C-8 5 µ, 250 x 15 mm and Waters X-Bridge C-18 2.5 µ, 150 x 4.6 mm for semipreparative and analytical runs respectively; Pump: Gilson gradient pump 322; UV/VIS-detector: Gilson 155; MS detector: Thermo Finnigan Surveyor MSQ; Gilson Fraction Collector FC204) using acetonitrile with 0.05% formic acid or triethylamine and deionized water with 0.05% formic acid or triethylamine as mobile phase. For preparative reversed phase purifications a VersaFlash™ system equipped with VersaPak™ C18 cartridges. MALDI-TOF MS was recorded in linear positive mode with α-cyano-4-hydroxycinnamic acid matrix (CHCA) or 2,5-dihydroxy benzoic acid (DHB) as matrix.

Synthesis of 1

3-thiopheneethanol (20.19 g, 157.5 mmol) was dissolved in DMF (100 mL) and the solution was cooled to -15 °C. NBS (22.4 g, 125.9 mmol) was added portion wise during one minute. The

solution was allowed to attain room temperature during 2 h, again cooled to -15 °C, more NBS (5.65 g, 31.7 mmol) was added portion wise during one minute, and the solution was allowed to attain room temperature. The reaction mixture was diluted with EtOAc, washed with brine, dried, filtered and concentrated. Purification by FC (DCM) and reversed phase VersaFlash™ gave **1** (73%) as colorless oil. R_f: 0.31 (toluene/ethyl acetate 4:1). ^1H NMR ($CDCl_3$) δ 2.84 (t, 2H, J = 6.6 Hz), 3.81 (t, 2H, J = 6.6 Hz), 6.82 (d, 1H, J = 5.2 Hz), 7.41 (d, 1H, J = 5.2 Hz); ^{13}C NMR ($CDCl_3$) δ 35.5, 62.4, 75.6, 128.5, 131.0, 143.3.

Synthesis of 2

A mixture of **1** (11.8 g, 57.0 mmol), 2.5- thiophenediboronic acid (4.80 g, 27.9 mmol), and K_2CO_3 (18.0 g, 130.2 mmol) in MeOH/toluene 1:1 (150 mL) was heated at 75 °C for 1 min. PEPPSI™-IPr catalyst (0.190 g, 0.280 mmol) was added and the mixture was heated at 75 °C for an additional 30 min. HOAc (conc.) and EtOAc were added and the mixture was washed with brine and water. The organic phase was dried, filtered and concentrated. Purification by reversed phase VersaFlash™ gave **2** (80%) as slightly yellow oil. R_f: 0.17 (toluene/ethyl acetate 2:1) Calcd mass for $C_{16}H_{16}O_2S_3$: $[M+H]^+$ 337.0; found 336.9. $[M+Na]^+$ 359.0; found 359.0. ^1H NMR ($CDCl_3$) δ 3.07 (t, 4H, J = 6.9 Hz), 3.89 (t, 4H, J = 6.9 Hz), 6.99 (d, 2H, J = 5.2 Hz), 7.10 (s, 2H), 7.22 (d, 2H, J = 5.2 Hz); ^{13}C NMR ($CDCl_3$) δ 32.7, 63.0, 124.6, 126.8, 130.2, 132.1, 135.4, 135.9.

Synthesis of 3

2 (328 mg, 0.975 mmol) was dissolved in DMF (3 mL) and the solution was cooled to -15 °C. NBS (351 mg, 1.97 mmol) was added portion wise during one minute. The solution was allowed to

attain room temperature during 2h. DCM and brine were added and the organic phase was separated, washed twice with 1M HCl, dried, filtered, and concentrated. The slightly crude yellow product **3** (99%) was used without further purification in the next step. ^{1}H NMR (300 MHz, $CDCl_3/CD_3OD$ 1:1) δ: 2.92 (t, *J* = 7.0 Hz, 4H), 3.74 (t, *J* = 7.0 Hz, 4H), 6.95 (s, 2H), 7.00 (s, 2H); ^{13}C NMR (75.5 MHz, $CDCl_3/CD_3OD$ 1:1) δ: 32.7, 62.2, 111.4, 127.4, 133.2, 133.4, 135.4, 137.1.

Synthesis of 4

To a solution of **3** (463 mg, 0.937 mmol) in MeOH/toluene 1:1 (5 mL) were added thiophene-2-boronic acid (475 mg, 3.71 mmol), and K_2CO_3 (647 mg, 4.68 mmol). The mixture was heated at 75 °C for 1 min and PEPPSI™-IPr catalyst (14 mg, 0.021 mmol) was added and the mixture was heated at 75 °C for an additional 20 min. HOAc (conc.) and EtOAc were added and the mixture was washed with brine and water. The organic phase was dried, filtered and concentrated. Short FC (ethyl acetate) gave product **4** (95%) as orange solid. ^{1}H NMR (300 MHz, DMSO-d6) δ 2.91 (t, *J* = 6.8 Hz, 4H), 3.68-3.77 (m, 4H), 4.83 (t, *J* = 5.2 Hz, 2H), 7.06-7.12 (m, 2H), 7.27 (s, 2H), 7.31 (d, *J* = 3.4 Hz, 2H), 7.51 (d, *J* = 5.0 Hz, 2H); ^{13}C NMR (75.5 MHz, DMSO-d6) δ 32.6, 60.7, 124.2, 125.7, 126.8, 127.4, 128.4, 129.0, 134.5, 134.8, 135.9, 137.7.

Synthesis of 5

Product **4** (0.049 g, 0.0979 mmol) and *p*-toluenesulphonyl chloride (0.056 g, 0.294 mmol) were added to a mixture of $CHCl_3$ (0.8 mL) and pyridine (0.2 mL). After 4h the solution was diluted with toluene and washed with HCl (1 M, aq.) and H_2O. FC (toluene) gave product **5** (84%) as yellow solid. ^{1}H NMR (300 MHz, $CDCl_3$) δ 2.32 (s, 6H), 3.11 (t, *J* = 6.6 Hz, 4H), 4.28 (t, *J* = 6.6 Hz, 4H), 6.86

(s, 2H), 7.01 (s, 2H), 7.03 (dd, J = 3.6, 5.1 Hz, 2H), 7.14 (dd, J = 1.1, 3.6 Hz, 2H), 7.20-7.26 (m, 6H), 7.69 (d, J = 8.3 Hz, 4H); ^{13}C NMR (75.5 MHz, $CDCl_3$) δ 21.6, 29.0, 69.4, 124.1, 125.0, 126.2, 127.0, 127.9, 128.1, 129.9, 131.0, 132.8, 134.0, 135.3, 136.5, 136.6, 144.9.

Synthesis of 6

5 (0.120 g, 0.148 mmol), Boc-L-Ser-OH (0.122 g, 0.593 mmol) and potassium carbonate (0.122 g, 0.890 mmol) were dissolved in 3 mL DMF and heated to 50 °C. After 1 day the reaction mixture was diluted with ethyl acetate and washed twice with HCl (1M, aq.) and three times with brine. The crude product was purified on FC (toluene/ethyl acetate 4:1) followed by HPLC ACN/H_2O 8:1->1:0 over 30 minutes to give product **6** in 45% yield as an orange solid. TLC (toluene/ethyl acetate 1:1) R_f: 0.30. ^{1}H NMR (300 MHz, $CDCl_3$) δ 1.43 (s, 18H), 3.17 (t, J = 7.1 Hz, 4H), 3.85 (dd, J = 3.6, 11.2 Hz, 2H), 3.92 (dd$J$ = 3.7, 11.2 Hz, 2H), 4.37-4.50 (m, 6H), 5.51 (d, J = 5.8 Hz, 2H), 7.02 (dd, J = 3.6, 5.1 Hz, 2H), 7.05 (s, 2H), 7.13 (s, 2H), 7.18 (dd, J = 1.1, 3.6 Hz, 2H), 7.23 (dd, J = 1.1, 5.1 Hz, 2H); ^{13}C NMR (75.5 MHz, $CDCl_3$) δ 28.4, 28.8, 56.0, 63.6, 65.1, 80.5, 124.2, 125.0, 126.4, 126.9, 128.3, 131.1, 134.7, 135.5, 136.4, 136.7, 155.9, 171.0.

Synthesis of p-HTE-Ser (7)

6 (0.048 g, 0.055 mmol) was dissolved in dichloromethane/TFA (4:1, 5 mL). After 2 h the solution was co-concentrated with toluene to give product **7** as red solid quantitively. ^{1}H NMR (300 MHz, DMSO-d6) δ 3.12 (t, 4H, J = 6.6 Hz), 3.69 (dd, 2H, J = 3.9, 11.6 Hz), 3.74 (dd, 2H, J = 4.3, 11.6 Hz), 3.93 (t, 3.9 Hz, 2H), 4.44 (t, J = 6.6 Hz, 4H), 7.11 (dd, J = 3.6, 5.1 Hz, 2H), 7.30 (s, 2H), 7.34 (dd, J =

1.1, 3.6 Hz, 2H), 7.35 (s, 2H), 7.55 (dd, *J* = 1.1, 5.1 Hz, 2H); ^{13}C NMR (75.5 MHz, DMSO-d6) δ 28.0, 54.8, 60.5, 64.6, 124.5, 126.0, 127.0, 127.3, 128.5, 129.4, 134.5, 135.2, 135.6, 135.7, 169.5

Synthesis of p-HTIm (8)

5 (116 mg, 0.143 mmol) and imidazole (500 mg, 7.34 mmol) were dissolved in MeCN (1.5 mL). The solution was heated at 65 ˚C for 2h and concentrated. FC (EtOAc + TEA and EtOAc/MeOH 4:1 + 1% TEA) gave crude compound that was further purified by gradient HPLC to provide p-HTIm (**8**) (51%) as orange solid. NMR was run on neutral compound, after which the sample was dissolved in 1M HCl, concentrated and dried to give a red-brown chloride salt. ^{1}H NMR (300 MHz, CD_3OD) δ 3.18 (t, *J* = 6.8 Hz, 4H), 4.27 (t, *J* = 6.8 Hz, 4H), 6.90 (app. t, *J* = 1.2 Hz, 2H), 6.92 (s, 2H), 6.93 (s, 2H), 6.97 (app. t, *J* = 1.3 Hz, 2H), 7.01 (dd, *J* = 3.6, 5.1 Hz, 2H), 7.18 (dd, *J* = 1.1, 3.6 Hz, 2H), 7.33 (dd, *J* = 1.1, 5.1 Hz, 2H), 7.46 (app. t, *J* = 1.1 Hz, 2H); ^{13}C NMR (75.5 MHz, CD_3OD) δ 31.9, 48.2, 120.7, 125.2, 126.2, 127.2 128.1, 129.1, 129.1, 131.8, 136.3, 137.0, 137.6, 137.8, 138.3.

Synthesis of p-HTMI (9)

5 (1.00 g, 1.24 mmol) was dissolved methylimidazole (4.75 mL) and DMF (1 mL). The mixture was heated at 75 ˚C for 4 hours and concentrated and co-concentrated with xylene to remove excess methylimidazole. Purification by gradient HPLC gave p-HTMI (**9**) (77%) as orange tosylate/formic acid salt. LC-MS calcd for $C_{32}H_{30}N_4S_5$: $[M]^{2+}$: 315.05; Found 315.10. ^{1}H NMR (300 MHz, D_2O, 35 °C) δ 2.22 (s, 4.4H, tosylate), 3.20 (t, *J* = 7.4 Hz, 4H), 3.91 (s, 6H), 4.33 (t, *J* = 7.4 Hz 4H), 7.05 (d, *J* = 4.2, 2H), 7.07 (s, 4H), 7,12 (d, *J* = 8.0 Hz, 2.9H, tosylate), 7.2 (d, *J* = 3.5 Hz, 2H), 7.28 (s, 2H), 7.35 (d, *J* = 5.1 Hz), 7,45 (s, 2H), 7.70 (d, *J* = 8.0 Hz, 3H, tosylate), 8.62 (s, 0.8H, formic acid), 8.66 (s, 2H); ^{13}C

NMR (75.5 MHz, D_2O, 35 °C) δ 20.9, 29.6, 36.1, 49.1, 122.2, 124.0, 124.6, 125.7, 125.9, 126.3, 127.5, 128.8, 129.3, 130.8, 134.5, 134.9, 136.1, 136.3, 136.6, 141.4, 141.4.

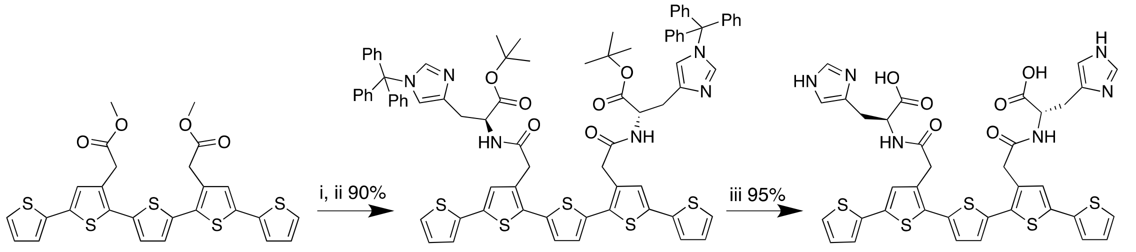

10, p-HTAM **11, p-HTA-His(1-Trt)-OtBu** **12, p-HTA-His**

Scheme 1. Reagents and conditions: (i) 1M NaOH, dioxane, H_2O; (ii) H-His(1-Trt)-OtBu, DIPEA, *O*-(7-azabenzotriazol-1-yl)-*N,N,N',N'*-tetramethyluronium hexafluorophosphate (HATU), DMF; (iii) Et_3SiH, TFA, DCM.

Synthesis of p-HTA-His(1-Trt)-OtBu (11)

To a solution of p-HTAM (**10**) (93 mg, 0.167 mmol) in dioxane (2 mL) and H_2O (0.5 mL) was added 1M NaOH (0.340 mL, 0.340 mmol). The solution was heated at 65 ˚C for 1 h, neutralized with 1M HCl, and the solvents were co-evaporated with toluene. The residual was dissolved in DMF (3 mL) and H-His(1-Trt)-OtBu (302 mg, 0.666 mmol) and DIPEA (0.100 mL, 1.04 mmol) were added. The temperature of the solution was lowered to 0 ˚C and *O*-(7-azabenzotriazol-1-yl)-*N,N,N',N'*-tetramethyluronium hexafluorophosphate (HATU) (190 mg, 0.500 mmol) was added. The solution was allowed to stir for 30 min at 0 °C and then for 4 h at room temperature. EtOAc, toluene and brine were added and the organic phase was separated and washed with saturated NH_4Cl (aq), saturated $NaHCO_3$ (aq) and H_2O, dried, filtered and concentrated. FC (toluene/EtOAc 1:1 + 1 % TEA gave p-HTA-His(1-Trt)-OtBu (**11**) (90 %) as yellowish oil. ^1H NMR (300 MHz, $CDCl_3$) δ 1.32 (s, 18H), 2.86-3.01 (m, 4H), 3.64 (d, *J* = 16.5 Hz, 2H), 3.70 (d, *J* = 16.5 Hz, 2H), 4.60-4.69 (m, 2H), 6.49

(d, *J* = 1.2 Hz, 2H), 6.92 (dd, *J* = 3.6, 5,1 Hz, 2H), 6.98-7.04 (m, 12H), 7.05 (dd, *J* = 1.1, 3.6 Hz, 2H), 7.12 (dd, *J* = 1.1, 5.1 Hz, 2H), 7.14-7.17 (m, 6H), 7.20-7.35 (m, 18H), 7.78-7.84 (b, 2H); ^{13}C NMR (75.5 MHz, $CDCl_3$) δ 28.2, 29.5, 37.4, 53.3, 75.3, 81.3, 119.3, 123.9, 124.7, 127.6, 127.9, 128.1, 129.9, 132.1, 132.2, 135.5, 136.1, 136.6, 137.0, 138.6, 142.4, 169.9, 170.3.

Synthesis of p-HTA-His (12)

pHTA-His(1-Trt)-OtBu (**11**) (35 mg, 0.025 mmol) was dissolved in DCM (1 mL) and Et_3SiH (0.035 mL, 0.414 mmol) was added. TFA (1 mL) was added and the solution was stirred for 3 h. The completeness of the reaction was validated by HPLC-MS. Solvents were co-evaporated with toluene. Purification by HPLC-MS gave p-HTA-His (**12**) (95 %) as orange solid. ^{1}H NMR (300 MHz, $(CD_3)_2SO$) δ 2.88-3.14 (m, 4H), 3.62 (s, 4H), 4.45-4.60 (m, 2H), 7.07 (s, 2H), 7.11 (dd, *J* = 3.6, 5.1 Hz, 2H), 7.23 (s, 2H), 7.29 (s, 2H), 7.30 (dd, *J* = 1.1, 3.6 Hz, 2H), 7.55 (dd, *J* = 1.1, 5.1 Hz, 2H), 8.13 (s, 2H), 8.52-8.59 (b, 2H).

Supplementary Figure legends

Supporting Figure S1: Structures of the LCOs evaluated.

Supporting Figure S2: The oligothiophene p-HTMI specifically detects embryonic neural stem and progenitor cells (NSPCs) *in vitro*. (**a**) The chemical structure of p-HTMI displays a methylated imidazole moiety. (**b**) Micrographs depicting staining of NSPCs and cells differentiated thereof. Staining obtained c:a 10 min after administration of p-HTMI (1:500). The staining showed a high signal-to-noise ratio and was predominantly cytoplasmic. (**c**) Micrograph depicting undifferentiated NSPCs 10 min after administration of p-HTA-His, an LCO with imidazole moieties lacking the methylation. (**d**) Two-photon microscopy confirmed that the p-HTMI labeling (green) was predominantly cytoplasmic. DAPI in blue labels the cell nucleus DNA. Ruler depicts pixels (arbitrary units). See also Supplementary Movie 1.

Supporting Figure S3: A different LCO, p-HTE-Ser, does not stain NSPCs, while a faint labeling can be detected in NSPC-derived early astrocytes and mesenchyme/smooth muscle like cells.

Supporting Figure S4. p-HTMI, but not p-HTE-Ser (P-HTES), efficiently and selectively labels NSPCs in mixed cell populations as assessed by FACS. p-HTE-Ser shows a similar distribution as control cells which has not been incubated with an LCO, whereas p-HTMI specifically detects the NSPCs, also in a mixed population of cells (log scale).

Supporting Figure S5. p-HTMI detects C6 glioma-derived and embryonic stem cell derived NSPCs. (**a**) Very few (<<1%) ESCs display staining by p-HTMI, whereas p-HTE-Ser stains >>90% of ESCs. (**b**) p-HTMI stains virtually 100% of ES cell-derived NSPCs. (**c**) Only small subsets of conventionally cultured C6 glioma cells with distinct morphology are stained by p-HTMI, whereas p-HTE-Ser stains >>90% of these cells. (**d**) Basically 100% of FGF2-exposed C6 glioma derived cells (C6DCs) are stained by p-HTMI, whereas p-HTE-ser does not stain these cells.

Supporting Figure S6. FACS analysis suggesting low specificity of CD44 in GSCs.

Supporting Movie M1: To investigate the subcellular localization of p-HTMI labeling, stacks of micrographs from two-photon microscopy were assembled creating a 3D picture demonstrating that the p-HTMI labeling was predominantly localized to the cytoplasm in NSPCs *in vitro*. Blue=DAPI, green=p-HTMI. Ruler depicts pixels (arbitrary units).

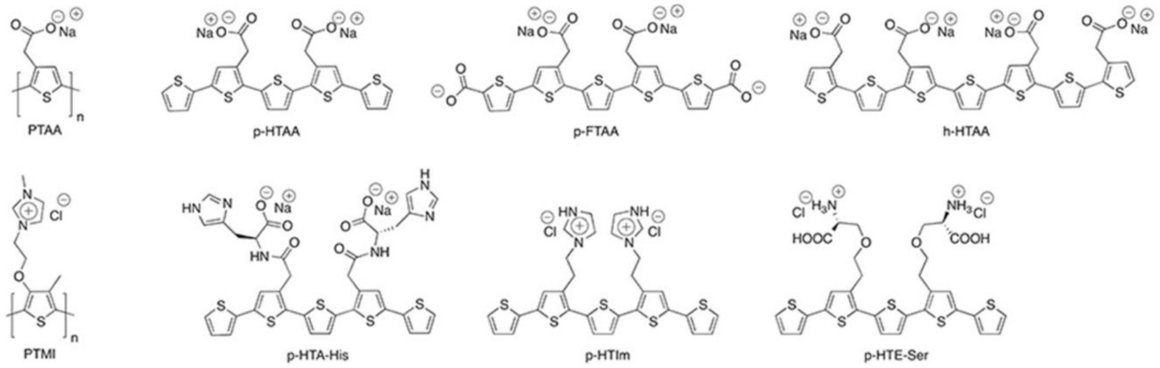

Ilkhanizadeh et al., supporting figure S1.

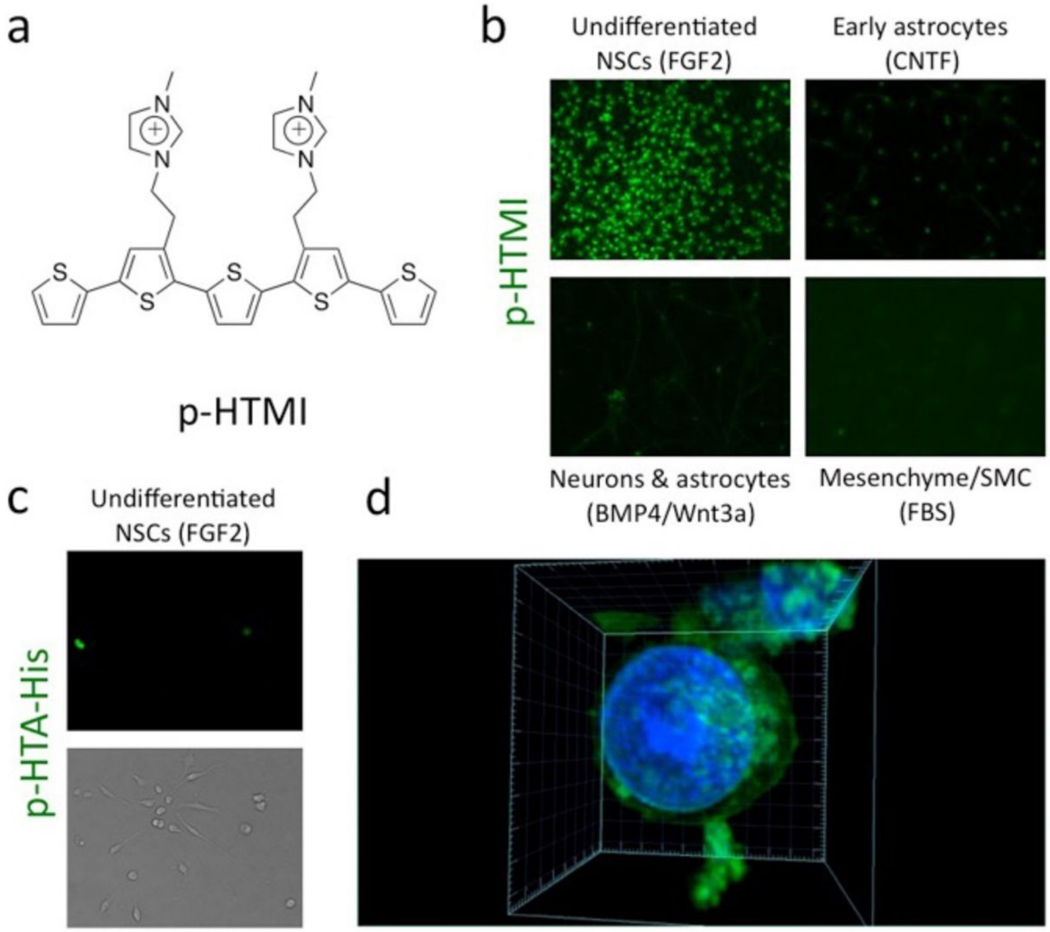

Ilkhanizadeh et al., supporting figure S2.

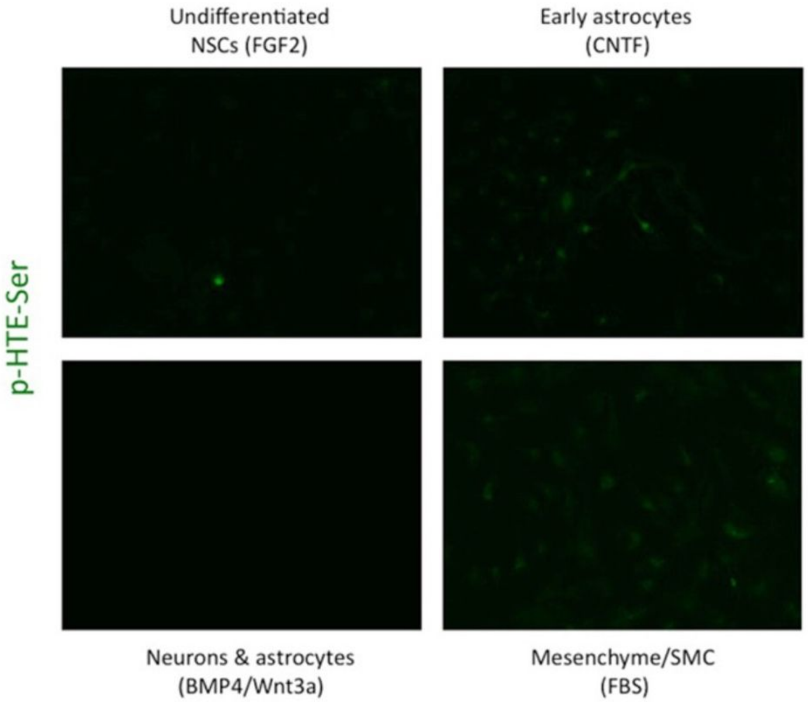

Ilkhanizadeh et al., supporting figure S3.

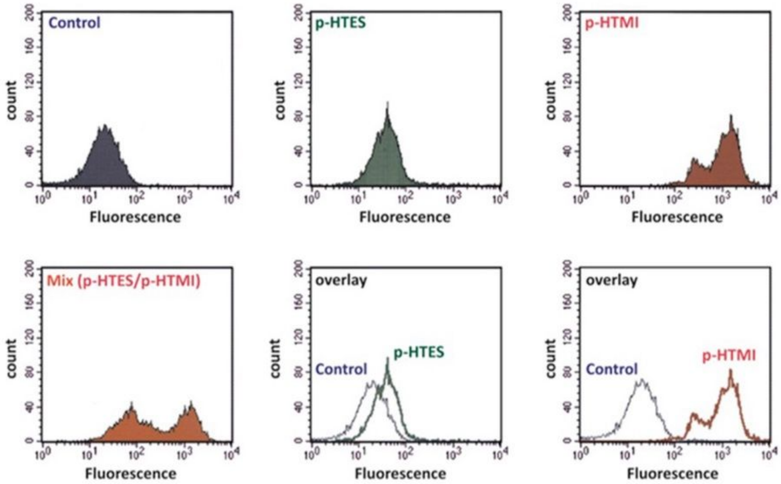

Ilkhanizadeh et al., supporting figure S4.

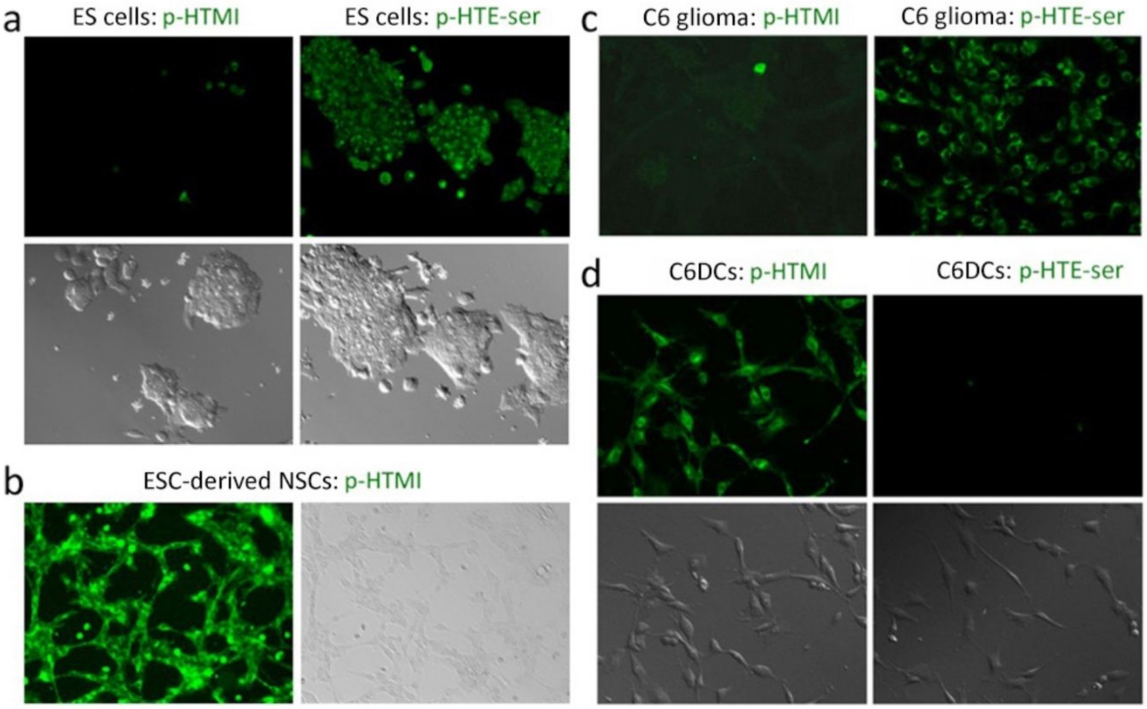

Ilkhanizadeh et al., supporting figure S5.

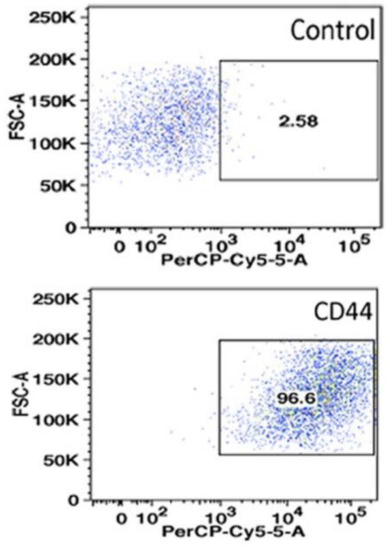

Ilkhanizadeh et al., supporting figure S6.
